# Surveillance on ESBL-*Escherichia coli* and Indicator ARG in Wastewater and Reclaimed Water of Four Regions of Spain: Impact of Different Disinfection Treatments

**DOI:** 10.3390/antibiotics12020400

**Published:** 2023-02-16

**Authors:** Márcia Oliveira, Pilar Truchado, Rebeca Cordero-García, María I. Gil, Manuel Abellán Soler, Amador Rancaño, Francisca García, Avelino Álvarez-Ordóñez, Ana Allende

**Affiliations:** 1 Department of Food Hygiene and Technology, Universidad de León, 24071 León, Spain; 2 Research Group on Microbiology and Quality of Fruit and Vegetables, Food Science and Technology Department, CEBAS-CSIC, 25, Espinardo, 30100 Murcia, Spain; 3 Entidad Regional de Saneamiento y Depuración de Murcia (ESAMUR), Avda. Juan Carlos I, s/n. Ed. Torre Jemeca, 30009 Murcia, Spain; 4 Acciona Agua, S.A.U., Avda. de Europa, 18, Parque Empresarial La Moraleja, 28108 Alcobendas, Spain; 5 Institute of Food Science and Technology, Universidad de León, 24007 León, Spain

**Keywords:** wastewater treatment plants (WWTPs), antibiotic-resistant bacteria (ARB), antibiotic resistance genes (ARGs), extended spectrum β-lactamase (ESBL)-producing *Escherichia coli*, reclaimed water

## Abstract

In the present study, the occurrence of indicator antibiotic-resistant bacteria (ARB) and antibiotic resistance genes (ARGs) both in the influent and the effluent of four Spanish wastewater treatment plants (WWTPs) was monitored for 12 months, and the susceptibility profiles of 89 recovered extended spectrum β-lactamase (ESBL)-producing *Escherichia coli* isolates were obtained against a wide range of antimicrobials. The aim of the study was to better understand whether the current wastewater treatment practices allow us to obtain safe reclaimed water mitigating the spread of ARB and ARGs to the environment. Results showed high concentrations of ESBL-producing *E. coli* as well as a high prevalence of a range of ARGs in the influent samples. The reclamation treatments implemented in the WWTPs were effective in reducing both the occurrence of ESBL *E. coli* and ARGs, although significant differences were observed among WWTPs. Despite these reductions in occurrence observed upon wastewater treatment, our findings suggest that WWTP effluents may represent an important source of ARGs, which could be transferred among environmental bacteria and disseminate antimicrobial resistance through the food chain. Remarkably, no major differences were observed in the susceptibility profiles of the ESBL *E. coli* isolated from influent and effluent waters, indicating that water treatments do not give rise to the emergence of new resistance phenotypes.

## 1. Introduction

The spread of antimicrobial resistant bacteria (ARB) and antimicrobial resistance genes (ARGs) is considered a major threat to human health, requiring an urgent action plan across every domain where the environment plays an important role [1]. A growing set of evidence suggests that the environment is a great contributor to the dissemination of ARB and ARGs [2,3,4]. Among these reservoirs, urban wastewater treatment plants (WWTPs) have been considered hotspots for antimicrobial resistance spread [2,5,6,7,8]. A meta-analysis study based on 57 works concluded that the prevalence of extended-spectrum β-lactamase (ESBL)-producing *Enterobacteriaceae* in wastewater has been increasing over the years [9]. Additionally, the same study reported a high prevalence for *bla_CTX-M_* genes in *Enterobacteriaceae* isolated from wastewater, accounting for approximately 67% of the ESBL genes detected, which is an important indicator of the spread of antimicrobial resistance.

Water scarcity is also recognized as one of the leading challenges of our time, with agricultural activity being one of its main drivers [10]. To overcome this global problem, the European Commission promotes an integrated water management approach, in which treated wastewater from urban WWTPs can represent an alternative water source to alleviate the demand for irrigation water [11]. Reclaimed water re-used for agriculture irrigation should meet some minimum quality requirements (MQR) to guarantee its safety, thus ensuring protection not only of the environment but also of human and animal health [11]. However, there is still a general unwillingness to accept reclaimed treated wastewater for irrigation partly due to the limited information existing on the efficacy of treatments applied in urban WWTPs to remove microbial contaminants, including ARB and ARGs, which may lead to their introduction in the farm fields. Some efforts have been made to study the elimination of microbial contaminants by various wastewater treatment processes [12]; however, the knowledge available on the selection and spread of ARB, as well as ARGs, in urban WWTPs is still limited. Some reports have recognized that conventional wastewater treatments are not completely effective in the removal or significant reduction of the potential risks to the environment posed by ARB and ARGs and that the application of advanced wastewater treatment technologies is necessary to control their occurrence and consequent release [13,14]. In this regard, in addition to the conventional secondary and tertiary wastewater treatment processes usually implemented in urban WWTPs, different methods have been recently studied and applied, including chlorination, adsorption and advanced oxidation processes, such as UV radiation, Fenton-reaction, solar-driven Fenton oxidation, photocatalytic oxidation, ozonation and ionizing radiation [15,16,17,18,19,20,21,22,23].

Some of the ARGs most commonly identified in urban WWTPs include integrase genes and genes associated with resistance to β-lactams, quinolones, sulfonamides, tetracyclines and macrolides [24]. Nevertheless, contradictory results have been reported regarding the occurrence of ARB and ARGs in WWTP effluents as compared to influents. Slightly higher antimicrobial resistance prevalence for enterococci and *Escherichia coli* [25] and *Enterobacteriaceae* [26] isolates were reported in WWTP effluents, as compared to influents. Similarly, an increased abundance of ARGs was found in WWTP effluents, as compared to influents, despite the significant reductions of about 99% obtained in the number of total bacteria [27,28,29]. Alexander et al. [27] reported a general increase in the abundance and release of three (*vanA*, *bla_VIM-1_* and *ampC*) out of four studied ARGs in the effluent of four WWTPs, as compared to the influents. However, the opposite trends have also been reported on other occasions. Yang et al. [30] reported a significant decrease in the abundance and diversity of ARGs revealed through the metagenomic sequencing of WWTP effluents. These authors reported lower relative abundances of tetracycline, multidrug and aminoglycoside resistance genes in the effluent as compared to influent samples. These findings agree with those of other studies, where a reduction of ARGs after the wastewater treatment process has also been detected [31,32]. Likewise, multi-drug resistant bacteria from different bacterial species found in the influent disappeared completely in post-treated effluents [33].

Based on published works, fecal indicators, including total coliforms, enterococci and *E. coli,* are the ARBs most frequently detected in urban WWTP samples. For example, Ferreira da Silva et al. [34] found high numbers of *E. coli* strains resistant to amoxicillin and tetracycline isolated from the treated effluent. Bacterial resistance to β-lactam antibiotics, classified as first-line antibiotics for common infections, has been reported worldwide, and the presence of ESBL-producing *E. coli* strains in effluent samples from urban WWTPs may contribute to its spread throughout the food chain, mainly through irrigation of fresh produce [35,36,37]. ESBL-producing *E. coli* are considered a One Health antimicrobial resistance (AMR) surveillance target due to several aspects, including their long use as a subject of environmental surveillance, as well as an indicator of faecal contamination in the microbial monitoring of food and water from WWTPs [38]. 

Previous studies focused on the surveillance of ARB and ARGs highlighted the need to implement regular surveillance and control measures, which may need to be appropriate for the geographic regions [3]. Additionally, there is an urgent need to validate the potential for wastewater treatments currently implemented in the WWTPs as an antibiotic resistance critical control point. The present study will therefore assess the occurrence of indicator ARB and ARGs both in the influent and the effluent of four Spanish WWTPs following different approaches for urban wastewater disinfection. Furthermore, the susceptibility profiles of 89 recovered ESBL-producing *E. coli* isolates against a wide range of antimicrobials were also examined in order to better understand whether the current wastewater treatment practices allow us to obtain safe reclaimed water mitigating the spread of ARB and ARGs to the environment.

## 2. Materials and Methods

### 2.1. Urban Wastewater Treatment Plants (WWTPs)

**Figure 1:**
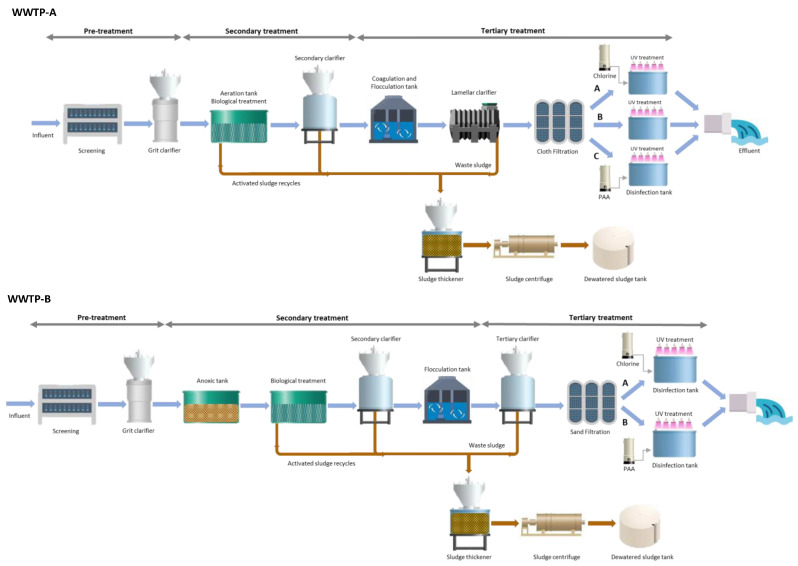

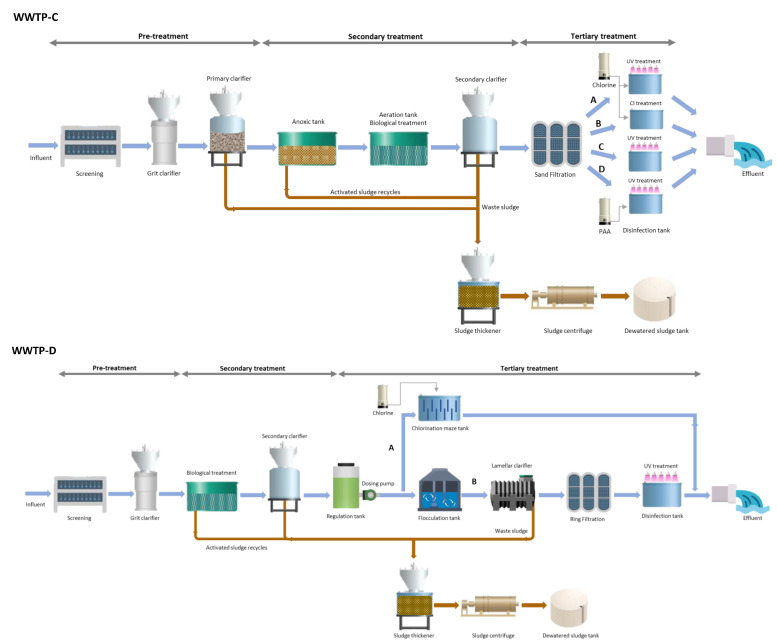
Schematic diagram of each WWTP including pre-, secondary and tertiary treatments applied.

Four urban WWTPs (plants A, B, C and D) with different treatment systems located in the south of Spain were used in this study. WWTP-A processed approximately 2750 m^3^ per day of mainly domestic wastewater from a population of about 35,252 inhabitants. WWTP-B treated approximately 4168 m^3^ per day of both domestic and industrial wastewater for a population of about 24,866 inhabitants. The amount of treated domestic and industrial wastewater from WWTP-C was about 1950 m^3^ per day for a population of about 12,062 inhabitants. In the case of WWTP-D, the amount of treated wastewater that flowed through was about 1850 m^3^ per day for a population of about 11,400 inhabitants. The schematic diagram of each WWTP is shown in Figure 1. In general, wastewater is primarily treated through aeration grit, including separation of suspended solids and particles, desanding-grease removal and a primary setting tank of different dimensions. The secondary treatment consists of a biological aerobic or anaerobic process in a secondary settling tank, including coagulation/flocculation and complementary lamellar clarification. In addition, disinfection processes are carried out with chlorine (Cl), ultraviolet radiation (UV) or the combination of chlorine/UV, or peracetic acid (PAA)/UV as tertiary treatments (Table 1). The disinfection treatments applied are adjusted based on the minimum effective doses required to meet microbiological quality standards. For those urban WWTPs that used chlorine, sodium hypochlorite with 10–20% active chlorine (NewChem, SL, Alicante, Spain) was added to maintain a residual concentration of free chlorine (FC) that varies between 0.1 and 3.0 mg/L. Combined treatments are performed by adding an aqueous chlorine solution or PAA into the tank, followed by a closed pipe or open channel UV disinfection system. UV doses ranged between 20–40 mJ/cm^2^. In the case of UV/PAA, a commercial solution of 15% PAA + 16% acetic acid + 24% hydrogen peroxide is used (Brenntag, Essen, Germany), reaching PAA concentrations between 3.0 and 5.0 mg/L.

### 2.2. Wastewater Sample Collection

The information regarding sampling time and tertiary treatment applied for each urban WWTP is shown in Table 1. Eight raw wastewater (influent) and tertiary effluent samples were collected monthly over a one-year period (June 2020 to May 2021) from the four urban WWTPs (plants A, B, C and D). Due to a mistake during sampling at WWTP-A, some samples were collected before the UV treatment was completed. The specific sampling times when this happened were June 2020, August 2020, November 2020, December 2020 and January to May 2021. Collected samples (1 L) in sterile polypropylene plastic bottles (Labbox Labware S.L., Barcelona, Spain) were stored under refrigeration conditions, transported within 2 h to the laboratory and stored at 4 °C until their analysis.

### 2.3. Enumeration and Isolation of Extended-Spectrum β-Lactamase-Producing E. coli (ESBL E. coli) 

A standard plate count method on CHROMagar ESBL (CHROMagar, Paris, France) plates was used for the enumeration of ESBL *E. coli* in all water samples. Depending on the expected bacterial concentration, serial decimal dilutions were prepared in sterile 0.2% buffered peptone water (BPW, Scharlab, Barcelona, Spain) and, subsequently, spread plating (0.1 mL), pour plating (1 mL) or membrane filtration (10 and 100 mL) methods were used. Samples were filtered through 0.45 μm cellulose nitrate membrane filters (Sartorius, Madrid, Spain) using a filter holder manifold (Millipore, Madrid, Spain). Plates were then incubated for 24 h at 37 °C, and dark pink-reddish colonies were counted. The analysis was performed in duplicate, and the results were expressed as cfu/100 mL. The detection limit (LOD) for ESBL *E. coli* counts in the raw water samples was 3.0 log cfu/100 mL (100 cfu/100 mL), while in the tertiary effluents, the LOD was 0 log cfu/100 mL (1 cfu/100 mL). When possible, five dark pink-reddish colonies on *E. coli* ESBL agar were picked from each positive sample and sub-cultured in brain infusion (BHI) at 37 °C for 24 h. After incubation, 1 mL of each culture was supplemented with 30% glycerol and kept at −20 °C until further analysis.

### 2.4. Wastewater DNA Extraction

Influent samples (10 mL) were concentrated by carrying out one centrifugation at 3000× *g* for 10 min. The supernatant was removed, and the pellet was resuspended in 1 mL of phosphate-buffered saline (PBS, Sigma-Aldrich, LS, USA). Subsequently, the resuspended pellet was centrifuged at 9000× *g*, 4 °C, for 10 min, and the supernatant was discarded. In the case of the effluent samples, water samples (100 mL each) were vacuum filtered through sterile cellulose nitrate filters (0.45 µm). Filters were placed in falcon tubes (50 mL) containing 20 mL of PBS (Sigma-Aldrich) supplemented with Tween 80 (1 mL/L; Sigma-Aldrich) and shaken in a vortex for 7 min. After that, the filters were removed, and the tubes were centrifuged at 3000× *g* for 10 min. Then, the supernatant was discarded, and the pellet was resuspended in PBS (1 mL). The resuspended pellet was concentrated by centrifugation (9000× *g*, 4 °C, 10 min). Influent and effluent pellets were kept at −20 °C until the genomic DNA extraction was performed. Genomic DNA was extracted and purified using the MasterPure™ kit (Lucigen, WI, USA) following the manufacturer’s instructions. An Implen NanoPhotometer N60/50 (Implen, Munich, Germany) was used to determine the concentration and purity of the DNA. All DNA samples were stored at −20 °C. 

### 2.5. Antimicrobial Resistance Genes (ARGs) Detection 

The presence of the ARGs *bla_CTX-M-G1_*, *bla_TEM_*, *catl*, *cmlA*, *qnrA*, *qnrB*, *sul1*, *sul2*, *tetA* and *tetB* was assessed by conventional polymerase chain reaction (PCR) using a T10Si 0 thermal cycler (Bio-Rad, CA, USA). Primer sequences and main PCR conditions are listed in Table 2. PCR reactions were performed in a 25 µL reaction mixture containing 5 µL of 5 × Flexi Buffer (Promega, Madison, WI, USA), 5 µL of 25 mM MgCl_2_, 0.50 µL of 10 mM dNTPs, 0.20 µL of 5 U/µL GoTaq G2 Hot Start Polymerase (Promega, Madison, WI, USA), DNAse free water and the primers listed in Table 2. In all cases, a non-template control (NTC) was included using 1 µL of DNAse free water instead of the DNA template. The PCR products were analyzed by electrophoresis of 2% agarose gels (SeaKem LE agarose, Lonza) supplemented with Red-dye staining (Biotium, Hayward, CA, USA) in Tris-borate-EDTA (TBE) buffer (89 mM Tris, 89 mM boric acid, 2.5 mM EDTA) at 80 V for 50–60 min. UV fluorescence emission was recorded and quantified by using ImageQuant™ LAS 500 (GE Healthcare Bio-Sciences AB).

### 2.6. Antibiotic Susceptibility Testing (AST)

A total of 89 ESBL *E. coli* isolates obtained from WWTP-A were subjected to AST. This treatment plant was selected considering that it contained the highest load of ESBL *E. coli* both in influent and effluent samples. The susceptibility of ESBL *E. coli* isolates to different antibiotics was determined by the microdilution method using Sensititre EUVSEC plates (Thermo Scientific, TREK Diagnostic Systems Ltd., East Grinstead, UK), according to the manufacturer’s instructions. Briefly, ESBL *E. coli* isolates cultured in Brain Heart Infusion broth (BHI, Merck, Germany) at 37 °C for 24 h were suspended in 10 mL of sterile water to reach a turbidity of McFarland 0.5. Subsequently, 20 µL of the ESBL *E. coli* suspension was transferred to 11 mL of Mueller-Hinton broth (Thermo Scientific, TREK Diagnostic Systems Ltd., East Grinstead, UK), and each well of the AST plate was filled in with 50 μL of the suspension using the Sensititre AIM Automated Inoculation Delivery System (Thermo Scientific, TREK Diagnostic Systems Ltd., East Grinstead, UK). After incubation at 37 °C for 24 h, the absence/presence of growth in each well was visually assessed to calculate the minimum inhibitory concentration of each antibiotic. The resistant or sensitive status of the isolate for each of the antimicrobials tested was determined considering the epidemiological cut-off value (ECOFF) of EUCAST (European Committee of Antimicrobial Susceptibility Testing). The antibiotics included in the AST panel were: sulfamethoxazole, trimethoprim, ciprofloxacin, tetracycline, meropenem, azithromycin, nalidixic acid, cefotaxime, chloramphenicol, tigecycline, ceftazidime, colistin, ampicillin and gentamicin.

### 2.7. Statistical Analysis

Non-zero microbial counts, evaluated by plating, were log-transformed (base-10) and stored along with zero counts (samples with undetected contamination) in an Excel spreadsheet (Microsoft Excel, 2016). For calculation and graphical representation of the median and interquartile range of microbial counts, only positive samples were included. Differences in log cfu/100 mL and prevalence (%) of ARGs were statistically analyzed after grouping them based on the WWTP (A, B, C, D) and the type of sample (influent and effluent) by using the *post hoc* Wilcoxon test, with significance established at *p* < 0.05. Boxplots were generated with *ggplot2* R-package. The antibiotic resistance profile of ESBL *E. coli* isolates to different groups of antimicrobials was represented in a heatmap generated with the *pheatmap* R-package. The statistical analyses were performed with R Studio (v 4.0.4).

## 3. Results and Discussion

The environment is thought to play an important role in the dissemination of antimicrobial resistance, with ESBL-producing *E. coli* being considered a relevant threat that particularly contributes to the horizontal transfer of critically important resistance determinants through the food chain. The current study demonstrates the frequent occurrence of ESBL-producing *E. coli* and of a range of indicator ARGs (including the ESBL genes *bla_CTX-M_* and *bla_TEM_*) both in influent and effluent waters from four Spanish WWTPs. Thus far, very few studies have examined the spatio-temporal distribution of ESBL-producing *E. coli* in urban WWTPs; therefore, it is difficult to find data for identical or even similar conditions across studies to compare the efficacy of tertiary treatments in their removal.

### 3.1. Occurrence of ESBL Producing E. coli in Wastewater Samples

**Figure 2:**
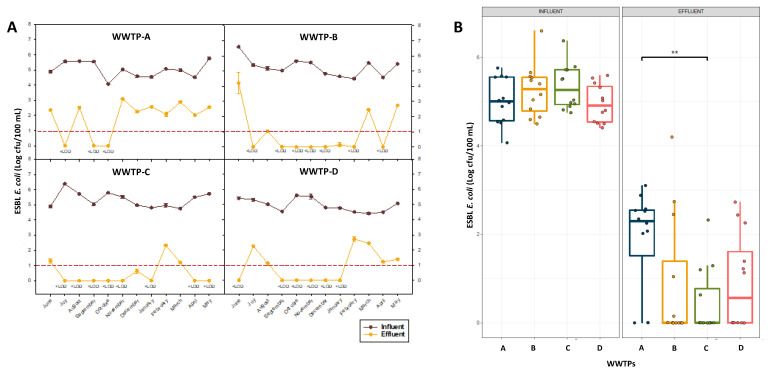
Spatio-temporal (**A**) and spatial (**B**) distribution of ESBL-producing *E. coli* (log cfu/100 mL) in influent and effluent samples for all four WWTPs between June 2020 and May 2021. In the boxplot, the solid horizontal line represents the median, and the box displays the 25–75% quartile range. Only significant *p* values (*p* < 0.05) obtained from the Wilcoxon signed-rank test analysis are indicated (**).

The spatio-temporal distribution of ESBL-producing *E. coli* counts in the four WWTPs studied during the one-year sampling period are presented in Figure 2A. Little variation, with similar medians among the different WWTPs in the influent counts, was observed (Figure 2B). ESBL-producing *E. coli* counts ranged from 4.1 to 5.8 log cfu/100 mL in WWTP-A, 4.5 to 6.6 log cfu/100 mL in WWTP-B, 4.8 to 6.4 log cfu/100 mL in WWTP-C and 4.4 to 5.6 log cfu/100 mL in WWTP-D. In general, the ESBL *E. coli* counts observed in the influent samples were quite high, with an overall mean of about 5.0 log units/100 mL, demonstrating that urban wastewater is an important reservoir of these bacteria. Similar concentrations of ESBL-producing *E. coli* in untreated urban wastewater were observed by Haberecht et al. [47], with 2.3 × 10^5^ cfu/100 mL, whereas Schmiege et al. [48] reported higher ESBL *E. coli* counts (7.5 × 10^5^ cfu/mL). Regarding the effluent samples in our study, significantly lower concentrations of ESBL-producing *E. coli* were observed (Figure 2B), with counts ranging from <LOD to 3.1 log cfu/100 mL in WWTP-A, <LOD to 4.2 log cfu/100 mL in WWTP-B, <LOD to 2.3 log cfu/100 mL in WWTP-C and <LOD to 2.7 log cfu/100 mL in WWTP-D. In general, the average concentration of ESBL-producing *E. coli* in the effluent water was below 1.0 log unit/100 mL, with most samples providing results below the detection limit (1 cfu/100 mL), with the exception of samples from WWTP-A, with an average count of approximately 2.0 log cfu/100 mL. It should be considered that in specific sampling times, effluent samples from the WWTP-A were collected before the UV treatment had been finalized, which affected the counts of ESBL-producing *E. coli*, particularly from November 2020 to May 2021. The fact that several sampling points showed results below the limit of detection (Figure 2A), mainly in WWTP-B, C and D, demonstrates that wastewater reclamation processes, including primary, secondary and tertiary treatments, were able to reduce ESBL *E. coli* counts in about 5.0 log units. These results highlight the efficacy of the water treatments employed, which are mainly UV light alone or in combination with chlorine and PAA, in reducing the ESBL *E. coli* load. Similarly, low levels of ESBL *E. coli* (around 2 cfu/mL) were previously reported by Raven et al. [49] in effluent waters after UV treatment as a disinfection method. Also, Bréchet et al. [50] observed lower concentrations of ESBL-producing *E. coli* in the treated water than in the untreated water (22 vs. 481 cfu/100 mL, respectively). In this case, the water was subjected to a typical flow treatment, including sedimentation, biological degradation and effluent polishing, without specification on the disinfection method used. Nzima et al. [51] determined the occurrence of ESBL-producing *E. coli* in surface water receiving an effluent discharge from a WWTP, and higher counts in the range of 2.5–3.3 log cfu/mL, were detected in all effluent samples when compared to our study. On the other hand, Solaiman et al. [52] reported that ESBL-producing *E. coli* were recovered at low prevalence in the ponds, rivers and reclaimed water examined.

Although a significant reduction of ESBL-producing *E. coli* counts was observed in effluent samples, as compared with influents, occasionally, higher counts were detected in different sampling months that exceeded 2.0 log units/100 mL. These outlier points were mostly observed in WWTP-B and D (Figure 2B), while samples from WWTP-A consistently showed high ESBL *E. coli* levels throughout most of the sampling period due to an incomplete application of the UV treatment. Nevertheless, significant differences (*p* < 0.05) were only observed between the counts in effluent samples from WWTP-A with respect to the samples taken from WWTP-C. It is worth mentioning that these occasional increases in ESBL *E. coli* counts in each of the WWTPs took place in different sampling months; hence they cannot be attributed to a seasonal influence. Seasonal differences were previously observed by Schmiege et al. [48], who reported higher ESBL *E. coli* counts during the winter season, indicating a higher antibiotic use against infections among the population in cold months. Similar trends were also documented in previous works [53,54]. The specific increases observed in our study can be due to different events, including factors related to the operation of the treatment plant or extrinsic factors, such as weather conditions or specific waste discharges.

### 3.2. Prevalence of Indicator ARGs in the Wastewater Collected from WWTPs

**Figure 3:**
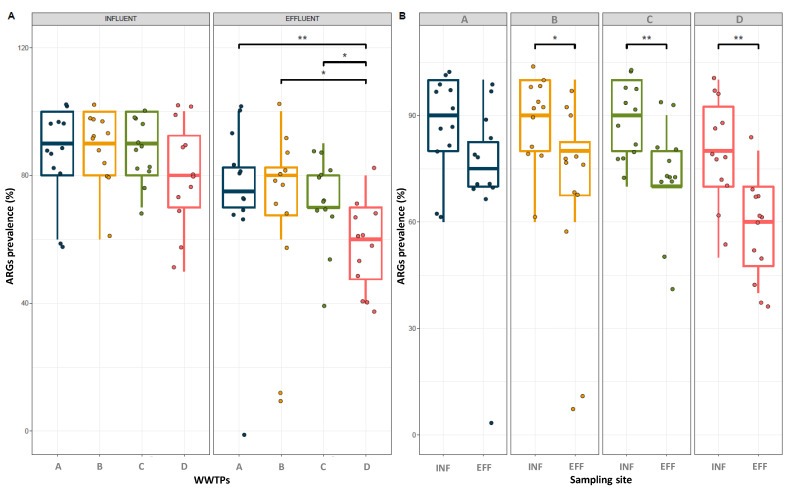
Boxplot of the prevalences (%) of ARGs detected in influent and effluent samples of each Urban Wastewater Treatment Plant (WWTP). (**A**) shows significant differences between WWTPs, and (**B**) shows significant differences between influent and effluent samples for each WWTP. Solid horizontal lines represent the median, and the boxes display the 25–75% quartile range. Only significant *p* values (*p* < 0.05) obtained from the Wilcoxon signed-rank test analysis are indicated (*, **).

The prevalence and distribution of ten ARGs associated with resistance to tetracycline (*tetA*, *tetB*), sulfonamides (*sul1*, *sul2*), quinolones (*qnrA*, *qnrB*), β-lactams (*bla_CTX-M-G1_*, *bla_TEM_*) and chloramphenicol (*catI*, *cmlA*) in the influent and effluent samples are shown in Table 3 and Figure 3. The ten studied ARGs were detected in the influent samples from all WWTPs with a very high prevalence, which ranged from 50 (six out of 12 samples) to 100%, with the only exception of the *qnrA* gene, which was detected in five out of 12 samples (41.67%) from WWTP-D (Table 3). The genes *cmlA* and *sul2,* associated with resistance to chloramphenicol and sulfonamides, respectively, were the most prevalent ARGs, with a prevalence of 100% in all WWTP influents. Moreover, the ARGs *bla_TEM_*, *catI*, *sul1* and *tetA* showed more than 80% prevalence in all WWTP influents. On the other extreme, ARGs associated with resistance to quinolones showed the lowest prevalence in all influent samples, which ranged from 41.67 to 66.67%. In Figure 3, the distribution of the prevalences obtained for all tested ARGs in influent and effluent samples for each WWTP showed statistical differences (*p* < 0.05) for effluent samples between WWTP-A, B and C and WWTP-D, with this latter one having, in general, the lowest ARG prevalences (Figure 3B).

A high abundance of ARGs in influent and effluent waters was previously reported after the extensive surveillance of 12 urban WWTPs located in seven countries (Portugal, Spain, Ireland, Cyprus, Germany, Finland and Norway) [3]. The authors observed a major abundance of ARGs associated with resistance against first-generation antibiotics in influent samples, including aminoglycosides (*aadA* and *strB*), β-lactams (*bla_GES_*, *bla_OXA_* and *bla_VEB_*), macrolides (*ereA* and *ermF*), sulfonamides (*sul1*), tetracyclines (*tetM* and *tetQ*) and multidrug resistance (*qacEdelta1* and *qacH*). Additionally, other genetic elements implicated in ARG transfer, such as *intI1*, *tnpA*, *Tp614*, *ISAba3*, *ISPps* and *ISSm2*, were also detected in all influents. On the other hand, ARGs of high clinical concern, like *bla_NDM-1_*, *bla_KPC_*, *bla_VIM_*, *bla_IMP_*, *mcr-1*, *mecA* and *vanA,* were barely detected in influents from the different countries. From the review published by Wang et al. [19] reporting data on ARGs abundance from 2007 to 2019, it was concluded that the most dominant ARGs frequently found in influent and effluent samples were *bla_CTX-M_*, *bla_TEM_*, *ermB*, *sul1*, *sul2*, *tetO*, *tetQ* and *tetW*.

Similar to the influent waters, a high prevalence of ARGs in all WWTP effluents was observed (Figure 3). All the ten ARGs considered were present in all the analyzed effluents, except in the case of the *qnrA* gene that was not detected in WWTP-C effluents (Table 3), illustrating that WWTPs can definitely be considered a potential hotspot for the dissemination of resistance genes to the environment, although the entire perspective of the dynamics of ARG spread through WWTPs is far from complete. In WWTP-B, C and D, a significant (*p* < 0.05) reduction in the prevalence of ARGs after tertiary water treatment was observed, while these reductions in prevalence were subtler and not statistically significant for WWTP-A (Figure 3B), probably due to the incomplete UV treatment applied in this WWTP. Reductions in ARG prevalence ranged from 8.33 to 58.33% depending on the WWTP and the ARG of concern (Table 3). For instance, the genes showing the highes decrease of prevalence (>50% reduction) were *bla_CTX-M-G1_*, *qnrA* and *tetA,* associated with resistance to β-lactams, quinolones and tetracycline, respectively. Noticeably, the gene *tetA* showed one of the highest prevalence values in influent samples and experienced one of the greatest reductions upon tertiary treatment of waters.

Nevertheless, this gene was still in high prevalence in WWTP-A and C effluents, showing values of 66.67% and 75%, respectively (Table 3). The gene *qnrA* gene showed the lowest prevalence in effluents of all ARGs considered in the study. It was not detected in WWTP-C effluents and showed a prevalence of 8.33, 25 and 33.33% in WWTP-D, A and B, respectively. Even though the occurrence of most of the ARGs decreased after treatment, ARGs were still discharged in effluent waters with a relatively high prevalence. Particularly, the *catI*, *cmlA*, *sul1*, *sul2*, *tetA, tetB* and *bla_TEM_* genes showed high prevalence in both influent (>83.33%) and effluent (>50%) samples. Similarly, *sul* genes appeared to be the most frequently detected in effluents from China, the USA and Sweden [55,56,57,58]. In our study, in some WWTPs, the prevalence of some of the studied ARGs remained almost unchanged. For example, the *cmlA*, *sul2* and *tetB* genes showed no removal at all in WWTP-C and D. Furthermore, *catI* and *cmlA* prevalence also remained unchanged after treatment in WWTP-B, and the same result was observed for the gene *bla_TEM_* in WWTP-A.

Among all treatment plants studied, the lowest prevalence values for all ten ARGs were observed in WWTP-D, which used UV light as a more frequent disinfection method during tertiary treatment, in some occasions combined with chlorine disinfection, indicating that when properly applied, the use of UV alone or combined with chlorine or other biocides can be an effective water disinfection treatment. However, the results previously reported regarding the use of UV light as a tertiary treatment to control the prevalence of ARGs are far from conclusive. For example, Auerbach et al. [59] observed no significant removal of *tet* genes and Ferro et al. [60] reported that the abundance of the *qnrS* gene remained unchanged and the abundance of the *bla_TEM_* gene increased after treatment. More recently, McConnell et al. [61] studied the efficacy of different UV doses on ARGs (*intl1*, *bla_CTX-M_*, *bla_TEM_*, *ermB*, *qnrS*, *sul1*, *sul2*, *tet(O)*, *mecA* and *vanA*) removal and they observed that the total ARG concentration decreased by 0.2 log copies/mL with a UV dose of 50 mJ/cm^2^ and around 0.6 log copies/mL with a higher UV dose of 250 mJ/cm^2^. Low levels of removal of *tet* and *sul* genes after UV disinfection were also observed in the past [58,59,62]. In disagreement with the results of some previous studies [32,57,63,64,65,66,67], an enrichment of ARGs after tertiary treatment was not observed in any of the four WWTPs here studied. For example, Mao et al. [57] observed a rise in the occurrence of twelve ARGs (*tetA*, *tetB*, *tetE*, *tetG*, *tetH*, *tetS*, *tetT*, *tetX*, *sul1*, *sul2*, *qnrB* and *ermC*) in the final effluents. Likewise, Marti et al. [66] also observed an increase in the relative abundance of almost all ARGs studied after wastewater treatment.

When comparing these results with those obtained on the occurrence of ESBL-producing *E. coli* in effluent samples, it can be seen that ARB and ARGs presented different responses to wastewater treatments. This is in agreement with results obtained in previous works where they observed that ARGs are more tolerant to wastewater treatments than ARB [24,60,68,69]. Moreover, these results can also be explained by the reduced detection sensitivity of bacterial enumeration techniques in environmental samples that may produce an underestimation of bacterial counts. The employment of qPCR techniques to quantify the absolute abundances of ARGs in WWTP samples will help to better assess the impact of ARGs released through effluents since the final risk is not only influenced by the presence of the genes but also by their concentration in the wastewaters. Additionally, through shotgun metagenomic DNA sequencing, Bengtsson-Palme et al. [70] investigated the relative abundances of ARGs in Sewage treatment plants (STPs). The authors observed a great reduction in the number of resistance genes per volume of water, although their relative abundance per bacterial 16S rRNA was only slightly decreased. It is worth mentioning that a few resistance genes, including the carbapenemase gene *bla_OXA-48_*, were enriched after the treatment processes in the STPs compared to the influent. However, the opposite trends have also been reported on other occasions. Yang et al. [30] reported a significant decrease in the abundance and diversity of ARGs revealed through the metagenomic sequencing of WWTP effluents. These authors reported lower relative abundances of tetracycline, multidrug and aminoglycoside resistance genes in the effluent as compared to the influent. These findings agree with those of other studies, where a reduction of ARGs after the wastewater treatment process has also been detected [31,32]. Likewise, multi-drug resistant bacteria from different bacterial species found in the influent disappeared completely in post-treated effluent in the study by Nimonkar et al. [33].

Thus, our findings, together with those previously reported, suggest that WWTP effluents may represent a source of ARGs, which could be transferred among environmental bacteria and disseminate antimicrobial resistance through the food chain when using reclaimed water for irrigation of crops. This is of utmost relevance, particularly in those areas where effluent water from the WWTPs is directly used as reclaimed water for the irrigation of crops. This is the case of the effluent from WWTPs C and D.

### 3.3. Antibiotic Resistance Profile of ESBL E. coli Isolates

**Figure 4:**
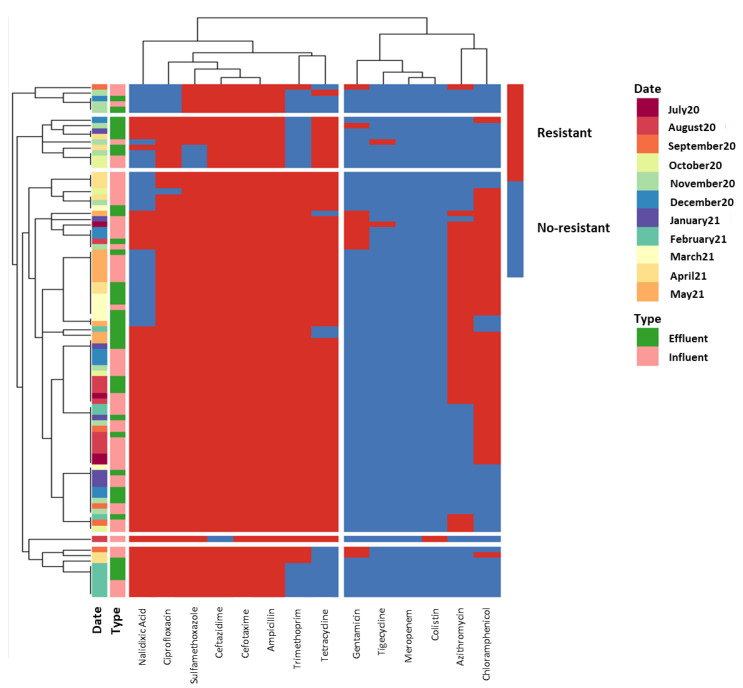
Heatmap showing the antibiotic resistance profile obtained for the ESBL producing *E. coli* isolates recovered from WWTP-A. ECOFF values from EUCAST served as threshold to classify the isolates (*n* = 89) into antibiotic resistant (in red) or susceptible (in blue).

A total of 89 ESBL *E. coli* isolates were recovered from influent and effluent waters of WWTP-A, the treatment plant showing the highest ESBL *E. coli* counts and ARGs prevalence in the effluents. Of these 89 ESBL *E. coli* strains, 37 were isolated from effluent samples. Figure 4 shows the resistance profile of these ESBL isolates to a broad range of antibiotics, including sulfamethoxazole, trimethoprim, ciprofloxacin, tetracycline, meropenem, azithromycin, nalidixic acid, cefotaxime, chloramphenicol, tigecycline, ceftazidime, colistin, ampicillin and gentamicin. The isolates showed resistance to a large number of antibiotics and can be considered multidrug-resistant (MDR) bacteria as they showed low susceptibility to at least one agent from three or more antimicrobial classes. While most isolates showed resistance to different β-lactam antibiotics (cefotaxime (100%), ceftazidime (99%), ampicillin (100%)), quinolones (ciprofloxacin (93%), nalidixic acid (66%)), tetracyclines (82%) and antifolate antimicrobials (sulfamethoxazole (96%), trimethoprim (79%)), almost all tested isolates (all the isolates from effluent samples) showed susceptibility to the action of last resort antimicrobials like meropenem, tigecycline and colistin. Interestingly, all isolates were susceptible to meropenem, and only one ESBL-producing *E. coli* isolate (from influent water) exhibited resistance to colistin but was susceptible to tigecycline. Resistance to tigecycline was observed in two isolates, also from influent samples. As shown in Figure 4, the clustering of isolates based on their antimicrobial susceptibility was mainly determined by the sampling date, while the type of sample (influent vs. effluent) had little influence. Similarly, Amador et al. [26] reported a high frequency of resistance in *Enterobacteriaceae* isolated from WWTP waters. They found resistance to several β-lactam antibiotics, tetracycline, ciprofloxacin and also to trimethoprim/sulfamethoxazole. Other studies have also reported antimicrobial resistance profiles for *E. coli* isolates originating from WWTPs [25,71]. Those isolates frequently presented resistance to sulfonamides, tetracyclines and aminopenicillins, while lower resistance rates were observed against quinolones. Noteworthy, the fact that no major differences were observed in the phenotypic profile of the ESBL *E. coli* isolated from influent and effluent waters indicates that water treatments do not give rise to the emergence of new resistance phenotypes. Furthermore, resistance to last-resort antibiotics (carbapenems, colistin, tigecycline) was very scarce, and when present, tertiary treatments were effective in removing them. However, this study has limitations, as only one indicator of ARB has been monitored. The prevalence of carbapenem resistance bacteria could have been higher if other bacterial species had been monitored, such as *Acinetobacter* or *Klebsiella*. Nevertheless, the presence of MDR bacteria in effluent waters is of particular concern considering the spread of antimicrobial resistance to the environment.

## 4. Conclusions

Based on the current literature, there remains a knowledge gap on how different wastewater treatments implemented in urban WWTPs may affect the occurrence or removal of ARB and ARGs in reclaimed water applied as irrigation water. The current study demonstrated the frequent occurrence of ESBL-producing *E. coli* and of a range of indicator ARGs, both in the influent and effluent waters from four Spanish WWTPs. The ESBL *E. coli* counts observed in the influent samples were quite high, while in the effluent samples, significantly lower concentrations were observed. The ten studied ARGs showed a high prevalence in the influent samples from all WWTPs, with the genes *cmlA* and *sul2* associated with resistance to chloramphenicol and sulfonamides, respectively, being the most prevalent ARGs. ARGs associated with resistance to quinolones showed the lowest prevalence in all influent samples. In general, all the ARGs were present in the effluent samples, except for the *qnrA* gene, illustrating that WWTPs can be considered a potential hotspot for the dissemination of resistance genes to the environment. However, the lower prevalence in the effluent samples indicates that, when properly applied, UV alone or combined with chlorine or other biocides can be an effective water disinfection treatment. The ESBL *E. coli* isolates showed resistance to a large number of antibiotics and can be considered multidrug-resistant (MDR) bacteria as they show low susceptibility to at least one agent from three or more antimicrobial classes. These findings demonstrate the relevance of urban wastewater as an important reservoir of these bacteria. However, it can be concluded that wastewater reclamation processes, including primary, secondary and particularly tertiary treatments, when adequately applied, are able to significantly reduce ESBL *E. coli* counts (in about 5.0 log units) and ARGs prevalence.

## Figures and Tables

**Table 1:** Urban wastewater Treatment Plants (WWTPs), sampling times, treated effluent flow and disinfection system: chlorine, peracetic acid (PAA), ultraviolet radiation (UV).

		Sampling Times											
Urban WWTPs	Parameters	06/2020	07/2020	08/2020	09/2020	10/2020	11/2020	12/2020	01/2021	02/2021	03/2021	04/2021	05/2021
**A**	**Treatment**	UV	UV	UV	Cl + UV	Cl + UV	UV	UV	UV	UV	PAA + UV	PAA + UV	PAA + UV
	**Effluent Flow (m^3^/day)**	_____	_____	2916	2600	2430	2029	2065	2126	1897	2900	2441	2441
**B**	**Treatment**	Cl + UV	Cl + UV	Cl + UV	Cl + UV	PAA + UV	PAA + UV	PAA + UV	PAA + UV	PAA + UV	PAA + UV	PAA + UV	PAA + UV
	**Effluent Flow (m^3^/day)**	4479	4180	4050	4065	3921	4150	3894	_____	_____	_____	_____	_____
**C**	**Treatment**	UV	Cl	UV	Cl + UV	UV	UV	PAA + UV	PAA + UV	PAA + UV	UV	UV	UV
	**Effluent Flow (m^3^/day)**	1700	1740	1533	1641	1673	1520	1529	1616	1714	2560	4284	2769
**D**	**Treatment**	Cl	UV	UV	Cl + UV	UV	UV	UV	UV	UV	UV	UV	UV
	**Effluent Flow (m^3^/day)**	1115	3366	_____	2272	1015	1895	2156	1914	_____	2504	2838	_____

**Table 2:** Conventional PCR primers used in this study.

Target Gene	Primer Sequence (5′ → 3′)		Concentration (µM)	Cycling Parameters	Amplicon Size (bp)	Reference
*bla* *_CTX-M-G1_*	FW	TTAGGAARTGTGCCGCTGYA	0.4	94 °C for 10 min; 35 cycles (94 °C for 40 s, 60 °C for 40 s, 72 °C for 1 min); 72 °C for 10 min	688	[39]
	RV	CGATATCGTTGGTGGTRCCAT				
*bla* *_TEM_*	FW	CATTTCCGTGTCGCCCTTATTC	0.4	94 °C for 10 min; 30 cycles (94 °C for 40 s, 60 °C for 40 s, 72 °C for 1 min); 72 °C for 10 min	800	[39]
	RV	CGTTCATCCATAGTTGCCTGAC				
*catA1*	FW	GGTGATATGGGATAGTGTT	0.4	95 °C for 7 min; 34 cycles (95 °C for 40 s, 55 °C for 1 min, 72 °C for 40 s); 72 °C for 5 min	349	[40]
	RV	CCATCACATACTGCATGATG				
*cmlA*	FW	GCCAGCAGTGCCGTTTAT	1	95 °C for 5 min; 35 cycles (95 °C for 30 s, 60 °C for 30 s, 72 °C for 30 s); 72 °C for 7 min	158	[41]
	RV	GGCCACCTCCCAGTAGAA				
*qnrA*	FW	GATAAAGTTTTTCAGCAAGAGG	0.5	95 °C for 7 min; 34 cycles (95 °C for 40 s, 55 °C for 1 min, 72 °C for 40 s); 72 °C for 5 min	543	[42]
	RV	ATCCAGATCGGCAAAGGTTA				
*qnrB*	FW	GATCGTGAAAGCCAGAAAGG	0.5	95 °C for 5 min; 38 cycles (95 °C for 40 s, 50 °C for 40 s, 72 °C for 40 s); 72 °C for 7 min	476	[42]
	RV	ATGAGCAACGATGCCTGGTA				
*sul1*	FW	TGAGATCAGACGTATTGCGC	1	95 °C for 4 min; 32 cycles (95 °C for 2 min, 52,7 °C for 2 min, 72 °C for 2 min); 72 °C for 10 min	400	[43]
	RV	TTGAAGGTTCGACAGCACGT				
*sul2*	FW	GCGCTCAAGGCAGATGGCATT	1	94 °C for 5 min; 30 cycles (94 °C for 15 s, 69 °C for 30 s, 72 °C for 60 s); 72 °C for 7 min	293	[44]
	RV	GCGTTTGATACCGGCACCCGT				
*tetA*	FW	GTAATTCTGAGCACTGTCGC	0.4	94 °C for 3 min; 25 cycles (94 °C for 1 min, 57 °C for 1 min, 72 °C for 1 min); 72 °C for 10 min	956	[45]
	RV	CTGCCTGGACAACATTGCTT				
*tetB*	FW	AAAACTTATTATATTATAGTG	0.4	94 °C for 3 min; 30 cycles (94 °C for 1 min, 52 °C for 1 min, 72 °C for 1 min); 72 °C for 10 min	169	[46]
	RV	TGGAGTATCAATAATATTCAC				

**Table 3:** Prevalence (%) of different ARGs detected in influent and effluent samples of each Urban Wastewater Treatment Plant (WWTP).

		Gene (nº Positive Samples/nº Total Samples)									
Urban WWTPs	Sampling Site	*bla* *_CTX-M-G1_*	*bla* *_TEM_*	*sul1*	*sul2*	*catI*	*cmlA*	*qnrA*	*qnrB*	*tetA*	*tetB*
**A**	**Influent**	83.33 (10/12)	91.67 (11/12)	83.33 (10/12)	100 (12/12)	100 (12/12)	100 (12/12)	50.00 (6/12)	66.67 (8/12)	100 (12/12)	100 (12/12)
	**Effluent**	66.67 (8/12)	91.67 (11/12)	75.00 (9/12)	91.67 (11/12)	91.67 (11/12)	91.67 (11/12)	25.00 (3/12)	58.33 (7/12)	66.67 (8/12)	83.33 (10/12)
**B**	**Influent**	75.00 (9/12)	100 (12/12)	91.67 (11/12)	100 (12/12)	83.33 (10/12)	100 (12/12)	66.67 (8/12)	66.67 (8/12)	100 (12/12)	91.67 (11/12)
	**Effluent**	58.33 (7/12)	83.33 (10/12)	75.00 (9/12)	83.33 (10/12)	83.33 (10/12)	100 (12/12)	33.33 (4/12)	58.33 (7/12)	33.33 (4/12)	75.00 (9/12)
**C**	**Influent**	75.00 (9/12)	100 (12/12)	100 (12/12)	100 (12/12)	100 (12/12)	100 (12/12)	50.00 (6/12)	75.00 (9/12)	100 (12/12)	75.00 (9/12)
	**Effluent**	41.67 (5/12)	91.67 (11/12)	75.00 (9/12)	100 (12/12)	91.67 (11/12)	100 (12/12)	0 (0/12)	66.67 (8/12)	75.00 (9/12)	75.00 (9/12)
**D**	**Influent**	75.00 (9/12)	91.67 (11/12)	91.67 (11/12)	100 (12/12)	100 (12/12)	100 (12/12)	41.67 (5/12)	66.67 (8/12)	83.33 (10/12)	58.33 (7/12)
	**Effluent**	16.67 (2/12)	50.00 (6/12)	83.33 (10/12)	100 (12/12)	66.67 (8/12)	100 (12/12)	8.33 (1/12)	58.33 (7/12)	33.33 (4/12)	58.33 (7/12)

## Data Availability

Not applicable.
